# Applications of Urinary Extracellular Vesicles in the Diagnosis and Active Surveillance of Prostate Cancer

**DOI:** 10.3390/cancers16091717

**Published:** 2024-04-28

**Authors:** Stephanie F. Smith, Daniel S. Brewer, Rachel Hurst, Colin S. Cooper

**Affiliations:** 1 Metabolic Health Research Centre, Norwich Medical School, University of East Anglia, Norwich Research Park, Norwich NR4 7TJ, UKcolin.cooper@uea.ac.uk (C.S.C.); 2 Department of Urology, Norfolk and Norwich University Hospitals, Norwich NR4 7UY, UK

**Keywords:** prostate cancer, extracellular vesicles, urinary biomarkers, active surveillance, diagnostics, RNA

## Abstract

**Simple Summary:**

Prostate cancer is the most common cancer among men. Diagnosis and risk stratification of prostate cancer can be challenging due to gaps in our understanding of the disease as well as the limitations of tests used in clinical practice. Extracellular vesicles are microscopic particles containing genetic material, proteins and other molecules that are released by cells. Urine contains extracellular vesicles which can originate from the prostate gland. This review addresses how extracellular vesicles are involved in the development of prostate cancer, as well as how urinary extracellular vesicles can be analysed to diagnose and monitor prostate cancer.

**Abstract:**

Prostate cancer is the most common non-cutaneous cancer among men in the UK, causing significant health and economic burdens. Diagnosis and risk prognostication can be challenging due to the genetic and clinical heterogeneity of prostate cancer as well as uncertainties in our knowledge of the underlying biology and natural history of disease development. Urinary extracellular vesicles (EVs) are microscopic, lipid bilayer defined particles released by cells that carry a variety of molecular cargoes including nucleic acids, proteins and other molecules. Urine is a plentiful source of prostate-derived EVs. In this narrative review, we summarise the evidence on the function of urinary EVs and their applications in the evolving field of prostate cancer diagnostics and active surveillance. EVs are implicated in the development of all hallmarks of prostate cancer, and this knowledge has been applied to the development of multiple diagnostic tests, which are largely based on RNA and miRNA. Common gene probes included in multi-probe tests include *PCA3* and *ERG*, and the miRNAs miR-21 and miR-141. The next decade will likely bring further improvements in the diagnostic accuracy of biomarkers as well as insights into molecular biological mechanisms of action that can be translated into opportunities in precision uro-oncology.

## 1. Introduction

Prostate cancer is the most common non-cutaneous cancer among men in the United Kingdom (UK) and the United States (US) [1,2]. It exhibits genetic, morphological and clinical heterogeneity [3]. Whilst some patients are found to have aggressive disease that rapidly metastasises, many others are diagnosed with low-risk indolent disease.

Rates of prostate cancer may be as high as 55% in men over the age of fifty years [4]; however, the majority of these men will live with undiagnosed, asymptomatic prostate cancer that causes no problems during their lifetime. Indeed, in populations screened with the prostate-specific antigen (PSA) test, only 1% of men diagnosed with prostate cancer and receiving no initial treatment had died from their disease after 10 years [5].

The side-effect profiles of radical prostate cancer treatments can negatively impact patient quality of life [6]. Risks of prostatectomy include urinary incontinence and erectile dysfunction as well as the risks associated with pelvic surgery and general anaesthesia; radical radiotherapy may result in lower urinary tract symptoms, urinary incontinence, diarrhoea, and bleeding. The overdiagnosis and overtreatment of indolent, low-risk prostate cancer is therefore particularly problematic in the context of low prostate cancer-specific mortality [7,8]. Active surveillance (AS), a period of close monitoring, is therefore offered to patients diagnosed with low-risk and favourable intermediate-risk prostate cancer to avoid the potential side effects of immediate radical treatments [9,10].

Despite significant developments in prostate cancer treatment over the last twenty years, accurate disease prognostication at the point of diagnosis and during active surveillance remains a clinical challenge. Serum PSA testing, multiparametric magnetic resonance imaging (mpMRI) and prostatic biopsy constitutes the standard diagnostic pathway for men with suspected prostate cancer in the National Health Service (NHS) in the UK [9].

There is a need for diagnostic tests with

(i) Higher specificity and sensitivity in distinguishing clinically significant prostate cancer from indolent disease [11];
(ii) Improved accuracy of diagnosis in the context of multifocal disease [12];
(iii) Feasibility for non-invasive and remote testing [13];
(iv) Improved cost-effectiveness [14];
(v) Improved environmental sustainability [15].

Clinical research priorities within the field of AS reflect the challenges in prostate cancer diagnostics more generally, namely the optimal selection of patients and accurate, timely identification of disease progression.

Extracellular vesicles (EVs) are membrane-bound envelopes containing molecular cargo (including proteins, nucleic acids, and lipids) [16] that can influence the cellular microenvironment [17]. The presence of EVs originating from all parts of the urogenital tract including the prostate gland have been demonstrated in the urine [18]. In this narrative review, we explore the functions of EVs in prostate cancer development and discuss the applications of EVs in urinary testing for the diagnosis and active surveillance of prostate cancer.

## 2. Prostate-Derived Extracellular Vesicles

### 2.1. Extracellular Vesicles: Definition and Terminology

There is great diversity in the composition and function of extracellular vesicles (EVs), and this is reflected in the literature, which shows great variation in the nomenclature used in studies involving EVs. In February 2024, the International Society for Extracellular Vesicles (ISEV) published an updated position statement summarising the consensus on the minimum information required for reporting of research on EVs. This document aims to increase rigor, reproducibility, and transparency during the design, execution, and reporting of EV studies [19]. It compiles feedback from over a thousand researchers worldwide. Within their guidelines, “extracellular vesicle” is defined as a generic term for particles that are released from cells, are delimited by a lipid bilayer, and cannot replicate on their own [19].

For subtyping, the ISEV recommend operational terms such as small and large extracellular vesicles, based on the diameter of the particles (e.g., small EVs which are <200 nm in diameter and large EVs which are >200 nm in diameter); however, caution is advised as the diameter can vary according to the characterisation method used. Other operational characteristics of EVs include their biochemical characteristics (e.g., CD63+ EVs) or descriptions of the conditions of their cell of origin (e.g., podocyte EVs, hypoxic EVs), as summarised in Table 1. The biogenesis-related terms ectosome (indicating origins in the plasma membrane) and exosomes (indicating origins in the endosomal system) are discouraged unless the subcellular origin of the EVs can be clearly demonstrated.

The last decade has seen significant progress in the field of EVs. There are now useful open access resources for researchers including Vesiclepedia, which is a community compendium for EVs [20], and ExoCarta, which is a database of EV contents that have been identified in different organisms [21].

### 2.2. Urine as a Source of Prostate-Derived Extracellular Vesicles

Urine has been a known source of EVs since the 1980s. Wiggins et al. published evidence suggesting the presence of urinary EVs through electron microscopy of normal urine back in 1986 [22]. Since then, numerous studies have been performed, detecting EVs and suggesting that EVs contain nucleic acids, proteins and transporters. These originate from all parts of the urogenital tract [23], including the kidneys, ureter, bladder, urethra, prostate (males), and vagina (females) [18]. In addition, urine also contains EVs of bacterial origin [24,25].

**Figure 1:**
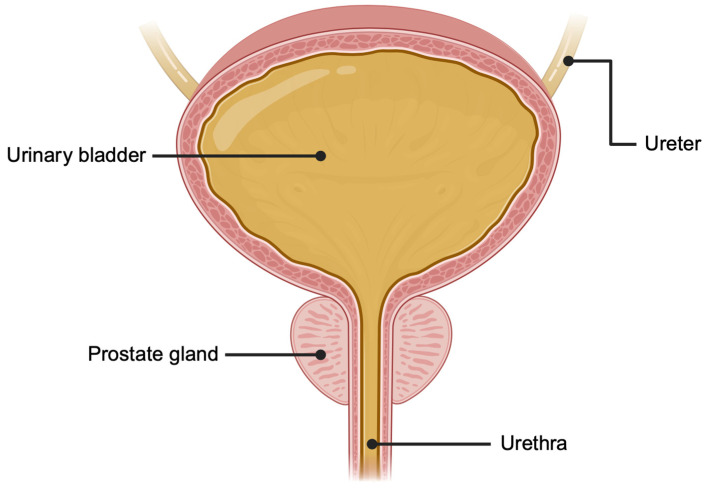
Schematic diagram (coronal view) of the male urogenital system illustrating the anatomical location of prostate gland inferior to the urinary bladder at the proximal urethra. Image created with biorender.com.

**Figure 2:**
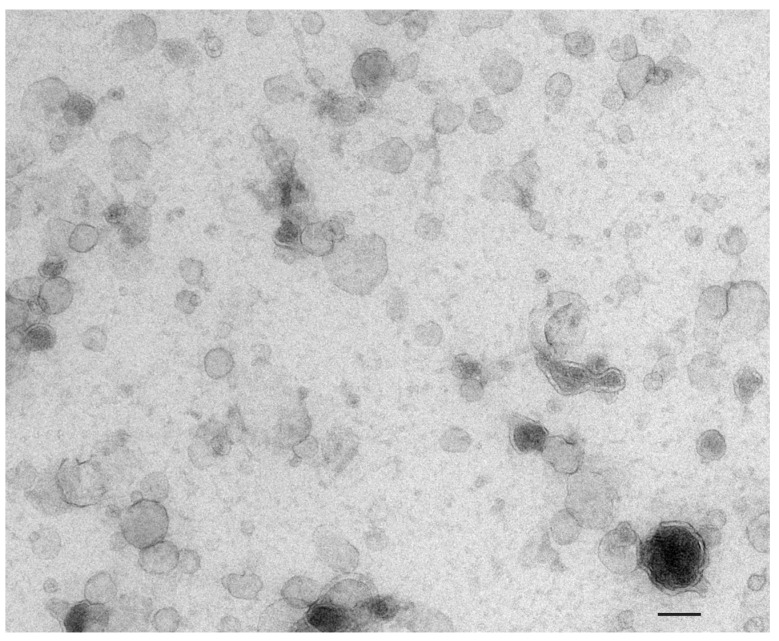
Transmission electron microscopy image of urinary extracellular vesicles. Scale bar indicates 100 nm.

Due to the anatomic relationship of the prostate gland and the urethra (Figure 1), prostatic secretions (containing EVs originating from the prostate gland) are found in the urine and can be expressed by a clinician performing digital rectal examination (DRE) prior to the patient micturating [26]. The DRE involves a clinician depressing the posterior aspect of the prostate gland with a finger via the rectum. Many studies of urinary biomarkers have been performed based on urine collection from the first urine voided after DRE [27]. Figure 2 demonstrates urinary EVs visualised on electron microscopy in a post-DRE urine sample.

In addition to patient discomfort and the requirement of a clinic appointment, a disadvantage of the post-DRE urine collection technique is that biomarker yields may vary significantly according to the technique of DRE used. This may depend on the clinician performing the DRE, reflecting real-life practice where techniques and clinician finger size may vary [28]. An alternative to post-DRE urine collection is collecting the first urine voided in the morning. Typically, these samples are more concentrated than random mid-stream urine collections. Without the necessity of DRE, this facilitates home (rather than clinic) collection, affording greater convenience for patients. Preservatives can stabilise urine for up to 6 months without diminishing urinary EV RNA yield or quality [28].

Urinary EV isolation techniques include centrifugation, filtration, immunoaffinity, and precipitation-based and microfluidics-based techniques [29]. These vary in equipment cost, time to perform isolation, ease of protocol, reproducibility, risk of contamination, and yield of EVs.

Utilising urinary biomarkers for prostate cancer diagnosis requires different considerations to serum-based biomarkers. Urine volumes and concentrations can vary considerably even within the same patient, both physiologically in health as well as pathologically in disease states and/or drug effects (e.g., diuretic use). Rather than using the volume of urine as a reference, biomarker ratios can be used instead. Much like the urine albumin to creatinine ratio (uACR) test used for evaluation of chronic kidney disease, prostate cancer diagnostics that detect RNA will typically utilise the detection of another transcript to generate an expression ratio or to “normalise” the result rather than reporting an absolute quantity, e.g., the ExoDx test detects *ERG* and *PCA3* relative to *SPDEF* [30].

### 2.3. Urinary Prostate-Derived Extracellular Vesicle Contents and Their Physiological Role

**Figure 3:**
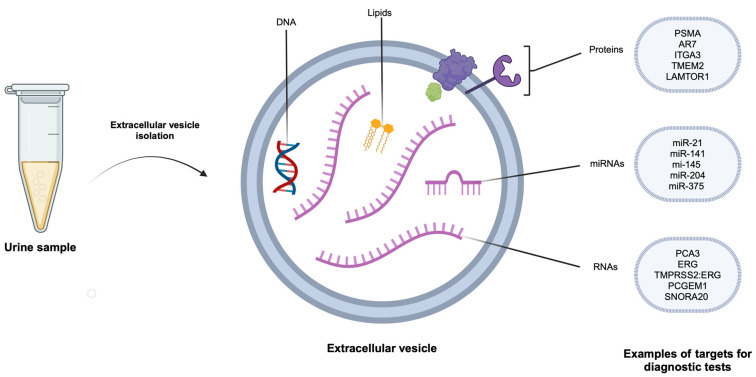
Schematic diagram of the contents of urinary extracellular vesicles from the prostate with examples of miRNA, RNA and protein-based diagnostic targets for prostate cancer. Image created with https://biorender.com (accessed on 14 April 2024).

Urinary prostate-derived EVs contain various molecular cargo, including nucleic acids (DNA, coding RNA, and non-coding RNAs including miRNAs), proteins, and lipids [18] as illustrated in Figure 3.

Secretory vesicles arising from the prostatic epithelia, termed “prostasomes”, were described by Brody et al. using electron microscopy back in 1983 [31] and subsequently characterised using immunohistochemical techniques by Nilsson et al. [32]. There is evidence that EVs are shed from both normal prostatic cells [31,32,33,34] and cancerous cells [35], as well as bacterial cells [19,36,37,38]. Our knowledge of the functions of EVs originating from the prostate gland in normal physiology is still underdeveloped, and our assumptions of their functionality are largely based upon studies of prostatic EVs in cancer; studies on the functions of prostate-derived EVs in the healthy state are sparse. One example of the role of prostatic EVs in health is their physiological role in facilitating fertilisation during reproduction [39,40]. Park et al. demonstrated that prostatic EVs transfer CD38 into sperm, which has a downstream effect in increasing sperm motility [41].

Chisholm et al. examined the contents of EVs extracted from healthy participants and patients with either organ-confined prostate cancer, extracapsular-extending prostate cancer, or seminal vesicle-invading prostate cancer. On proteomic analysis, they found distinct groups of proteins differently expressed between the groups of patients and healthy subjects [42]. EVs are more abundant in prostate cancer patients as compared to healthy individuals as well as being secreted in higher quantities by malignant cells than normal prostate epithelia [43,44,45,46]. The concentration of EVs correlated with the grade of prostate cancer [47].

## 3. Extracellular Vesicle Functions in Prostate Cancer

Historical experiments on sheep reticulocyte maturation suggested that the primary function of EVs was in the clearance or externalisation of cellular waste such as obsolete membrane proteins [48]. Since then, a growing body of evidence suggests that EVs play an important role in intercellular communication, which is dynamic and multidirectional [49]. Therefore, the functions of EVs are context-dependent (varying with the cell of origin, the EV content, the recipient cell, and the extracellular microenvironment).

In the context of prostate cancer, EVs can be considered to carry the carcinogenic propaganda that incites the development of the hallmarks of cancer: sustaining proliferative signalling, enabling replicative immortality, evading growth suppression, activating invasion and metastasis, inducing angiogenesis, causing genomic instability and mutation, resisting cell death, deregulating cellular metabolism, avoiding immune destruction, and inducing tumour-promoting inflammation [50]. EVs are also implicated in the mechanism by which the polymorphic urinary microbiome acts as an enabling agent to facilitate hallmark acquisition [51]. The influence of EVs in enabling each of these hallmarks of prostate cancer is explored below.

### 3.1. Sustaining Proliferative Signalling

Dysregulation of the cell growth and division cycle enables sustained cellular proliferation in carcinogenesis. The binding of growth factors to cell-surface receptors containing intracellular tyrosine kinase domains is implicated in this process [50].

EVs can transfer receptors from prostate cancer cells to other surrounding cells. Kharmate et al. demonstrated the presence of epidermal growth factor receptor (EGFR) in EVs isolated from prostate cancer lines [43]. *EGFR* is an oncogene which, upon ligand binding, activates intracellular signalling pathways that result in cellular proliferation [52], and its overexpression has been implicated in driving the progression of multiple cancer types including prostate, breast, lung, and glioblastoma [53]. In prostate cancer, *EGFR* expression is associated with higher Gleason scores and with time to biochemical relapse following radical treatment [54]. *Src*, *IGFR1R* and *FAK*, which are also implicated in proliferation pathways, have been demonstrated to be enriched in prostate cancer EVs [55], suggesting that Evs are disseminators of the molecular drivers of cellular proliferation.

The androgen receptor (AR) regulates transcription, genomic stability and DNA repair [56] and is of clinical relevance in prostate cancer as a therapeutic target used in androgen deprivation therapy. *AR* expression is a key driver in the development and progression of prostate cancer, and EVs have been implicated in transferring ARs between cells. Read et al. found that *AR* and its mutant variant, *Arv7*, were secreted in EVs derived from prostate cancer cell lines and transported to the nucleus of AR-null cells [57]. The transported EV-derived *AR* was then able to bind the androgen-responsive promoter region of PSA and recruit RNA Polymerase II, ultimately enhancing the proliferation of these acceptor cells in the absence of androgen. By injecting EVs into the prostate glands of male mice, they demonstrated nuclear localisation of the AR *in vivo*. This may provide a molecular mechanism for the switch from hormone-sensitive to hormone-resistant prostate cancer.

Prostate EVs have also been found to contain and transfer RNA that influences proliferative signalling pathways in recipient cells. Zheng et al. demonstrated that the circular RNA *circSLC19A1* was highly expressed in EVs secreted by prostate cancer cells, which resulted in the promotion of PCa proliferation by absorbing miR-497 to upregulate expression of septin 2, a GTP-binding protein, with downstream effects mediated through the ERK1/2 pathway [58]. Silencing *circSLC19A1* inhibited the proliferation, migration, and invasion of prostate cancer cells (DU 145 and PC3 cells) [59].

### 3.2. Enabling Replicative Immortality

Unlimited replicative potential enables cancer cells to develop into macroscopic tumours, in contrast to normal cells, which are limited by a fixed number of cell growth and division cycles [50]. The uncontrolled activation of telomerase plays a fundamental role in enabling replicative immortality through the elongation of telomeric DNA. In addition, the protein subunit human telomerase reverse transcriptase (TERT) is implicated in amplification of the Wnt pathway signalling, which has a role in prostate cancer development and progression [60].

Cell-free circulating plasma *hTERT* mRNA is associated with characteristics of poor prognosis in prostate cancer patients [61]. Yields of free circulating *hTERT* mRNA, however, are low and unstable, restricting its utility as a diagnostic biomarker. Goldvaser et al. therefore evaluated telomerase mRNA derived from EVs and found that 62% of cancer patients expressed EV-derived telomerase mRNA, as compared to none in healthy controls [62]. Despite the small sample size of prostate cancer patients in the study (3 out of the 133 cancer patients had prostate cancer), this shines a light on the molecular mechanisms of carcinogenesis as well as highlighting the potential utility of EV *hTERT* mRNA as a prostate cancer biomarker. Interestingly, in one prostate cancer patient (a 92-year-old male), they performed serial sampling and demonstrated that the transcript levels increased from 1.5 at diagnosis to 10.5 one month later, highlighting their potential application in disease monitoring.

### 3.3. Evading Growth Suppression

EVs have been implicated in the mechanisms by which prostate cancer cells evade growth suppression signalling. Important tumour suppressors include TP53 and PTEN. TP53 can stop progression of the cell cycle in response to cellular stress [50] and is lost in approximately one quarter of primary prostate cancers [63]. PTEN also regulates the cell cycle [64] and is lost or mutated in approximately half of metastatic castrate-resistant prostate cancers [65].

PTEN can be transferred between prostate cells via EVs [66]. Chowdhury et al. demonstrated EV-mediated delivery of catalytically active PTEN to PC3 cells using Western blot and fluorescence microscopy. They found that delivery of normal PTEN via EV to PTEN-deficient PC3 prostate cancer cells resulted in growth arrest alongside growth arrest when PC3 cells were exposed to EVs isolated from senescent cells [67]. Delivery of PTEN represents an exciting potential future cancer therapeutic strategy; proof of concept has been achieved in vivo, whereby *PTEN* mRNA delivery via nanoparticles resulted in restoration of tumour growth suppression in a murine model [65], and human studies are eagerly awaited.

MicroRNAs, transferred by EVs, can also downregulate these tumour suppressor proteins, leading to evasion of growth suppression. For example, PTEN can be downregulated by the action of microRNA miR-106a. Lu et al. showed that overexpression of miR-106a promotes the growth of prostate cancer cell line PC3 [68]. Whilst studies of EV-mediated transfer of miR-106a have not yet been performed in prostate cancer cells, EV transfer of miR-106a has been demonstrated to contribute to tumorigenesis in nasopharyngeal carcinoma [69].

The microRNA miR-27a, transferred from prostate fibroblasts (PSC27) by EVs, has been shown to restrain growth of prostate cancer PC3 cells [70]. miR-27a reduced the expression of TP53, and miR-27a expression increased with chemotherapeutic agent treatment (cisplatin, doxorubicin, and docetaxel) [70]. This suggests an EV-mediated means by which the microenvironment can influence prostate cell properties and accelerate carcinogenesis through evasion of growth suppression mechanisms.

### 3.4. Activating Invasion and Metastasis

Perhaps the most important feature differentiating cancerous from normal prostate epithelial cells is their ability to invade and, ultimately, metastasise to distant anatomical sites. The epithelial–mesenchymal transition (EMT) describes the process by which prostatic epithelial cells change to a mesenchymal phenotype with increased ability to invade and migrate [71]. EVs have been found to modulate this transition by transferring nucleic acids and proteins, with consequences for cellular adhesion, cytokine signalling pathways, and the response to environmental or systemic factors (e.g., adiposity, hypoxia) [72,73,74,75,76,77,78,79].

Using a multi-well cell invasion assay, Corcoran et al. demonstrated that the prostate cancer cell line DU145 underwent a significant increase in cell invasion when EVs isolated from the serum of prostate cancer patients were applied in vitro (as compared to the application of EVs from age-matched healthy controls) [72]. It is thought that the mechanism of action of the EVs is by transfer of nucleic acids and proteins that on internalisation induce a change in phenotype in the recipient cells. Brzozowski et al. found that addition of prostate cancer cell line EVs to prostate epithelial cell lines (RWPE1) in vitro altered the expression of two tetraspanins (CD9 and CD151), which are molecular scaffolds with roles in cell adhesion, and resulted in increased cell invasion and migration [73]. They also observed that alteration of the CD9 and CD151 in the donor cells changed the proteome of the EVs that they produced, providing further evidence of the importance of EVs in reflecting the dynamic state of the cell of origin and their role in intercellular communication.

Abnormalities in the transforming growth factor-β (TGF-β) signalling pathway and the androgen signalling pathway can lead to EMT [74]. El-Sayed et al. evaluated the effects of EVs isolated from a prostate cancer cell line which had mesenchymal-like properties (Mes-PCa) on androgen-dependent epithelial prostate cancer cells. They found that the Mes-PCa EVs promoted mesenchymal feature development in the recipient cells, modulated androgen receptor signalling, and activated the TGF-β pathway. This resulted in the recipient cells displaying enhanced invasive features [75], and suggested that EVs play an important role in prostate cancer progression and resistance to hormone treatment.

EV-transmitted mRNAs are also important in facilitating EMT. The presence of *TMPRSS2:ERG* mRNA has been demonstrated in urinary EVs with good correlation to prostatic tissue expression [80]. *TMPRSS2:ERG* gene fusion, which is reported to occur in between 20–80% of prostate cancer [81,82], leads to changes promoting EMT, as demonstrated in an in vitro prostate cancer model consisting of immortalised prostate epithelial cells in culture [83]. Leshem et al. observed that in their prostate cancer model consisting of a prostate cancer cell line that expressed the *TMPRSS2:ERG* gene fusion, the cells developed fibroblastic-like morphological characteristics and had a higher degree of adherence between their neighbouring cells [83]. They noted on microarray, QRT-PCR, and immunofluorescence studies that these cells also had a reduction in the levels of *CDH1* mRNA. There was a reduction in the cell surface protein E-cadherin, which is a key event during EMT [83].

EV-transmitted small microRNAs have also been demonstrated to play a role in invasion and metastasis in prostate cancer. Shin et al. demonstrated that urinary EV miR-142-3p was associated with prostate cancer metastasis [84]. The mechanism underlying this is likely due to regulation of Forkhead box transcription factor O1 (FOXO1); miR-142-3p is negatively correlated with FOXO1, and Tan et al. found that miR-142-3p knockout impaired tumour growth in male mice [85]. Other microRNAs of importance in EMT include miR-21, which can regulate cell invasiveness through control of RECK, a key inhibitor of several metalloproteinases [86]. In addition, miR-210 can activate NF-kappa B signalling and thereby promote epithelial mesenchymal transition, invasion, and migration [87].

The microenvironmental conditions and stresses can influence the ability of EVs to induce phenotypic changes in prostatic cells that lead to invasion and metastasis. Resistin is a secretory factor that is produced by adipose tissue and is known to influence other signalling pathways implicated in cancer such as the Toll-like receptor 4 (TL4) and the PI3K/Akt/NFκB pathways. Oregel-Cortez et al. demonstrated that EVs isolated from resistin-treated prostate cancer PC3 cells were more invasive due to increased p-FAK levels as well as increased secretion of the metalloproteinases MMP-2 and MMP-9 [76] in vitro. Phosphorylated FAK is associated with metastasis, and the metalloproteinases MMP-2 and MMP-9 have roles in degrading the extracellular matrix [77].

Another example of EVs acting as mediators in driving carcinogenic changes in prostatic cells influenced by the microenvironment is in hypoxia. Ramteke et al. exposed LNCaP and PC3 cells to hypoxic conditions (1% O_2_) and isolated their EVs for analysis [78]. They found that the hypoxic cells had smaller sized EVs than normoxic cells; higher levels of CD63, CD81, heat shock proteins (HSP90 and HSP70), and Annexin II; and higher metalloproteinase activity. Co-culturing these EVs with LNCaP and PC3 cells increased both their invasiveness and motility. Deep et al. went on to further investigate the effects of hypoxic EVs in a murine in vivo model [79]. Histological analysis demonstrated that the hypoxic EVs enhanced *MMP2*, *MMP9*, fibronectin and collagen in multiple sites (prostate gland, seminal vesicles, bladder, brain, lymph nodes, lung, heart, liver, spleen, kidneys, bone), suggesting that EVs have an important role in influencing the microenvironment at distal sites of metastasis.

EVs are also involved in both the development and optimisation of pre-metastatic niches (PMNs). Bone is a common site of metastasis in prostate cancer, with osteoblastic-type (sclerotic) lesions. Many men who die from prostate cancer are found to have bone metastases upon autopsy [88]. It is thought that long-distance signalling between the primary site and distal sites of future metastasis leads to the development of PMNs that promote and augment the growth of disseminated tumour cells during metastasis [89]. EVs produced by prostate cancer cells can mediate PMN formation, with prostate cancer-derived EVs frequently targeting bone marrow-derived cells. Henrich et al. characterised EV-mediated communication between prostate cancer cells and the bone marrow, identifying EV uptake by bone marrow myeloid cells and activation of NFκB [90]. They found that fluorescent-labelled EVs from enzalutamide-resistant prostate cancer cells were more robustly taken up by bone marrow macrophages in a murine in vivo model, approximately five-fold more than EVs from control cells (normal prostate epithelium). This effect was shown to be cholesterol-dependent: reducing myeloid cell cholesterol prevented EV uptake and abolished NFκB activity and osteoclast differentiation, reducing the metastatic burden by 77%.

EV-derived microRNAs are also implicated in PMN formation. Hashimoto et al. found that hsa-miR-940, secreted by prostate cancer cells via EVs, promoted osteogenic differentiation of human mesenchymal stem cells in vitro as well as inducing osteoblastic lesions in the bone metastatic microenvironment in vivo in a murine model [91].

### 3.5. Inducing Angiogenesis

EVs can mediate pro-angiogenic effects at the site of the primary prostate tumour as well as pro-angiogenic effects at metastatic sites.

Luo et al. demonstrated that there were higher levels of EV-derived PGAM1 in the plasma of patients with metastatic prostate cancer [92]. PGAM1 was subsequently demonstrated to promote angiogenesis by binding to γ-actin (ACTG1), which promoted podosome formation and neovascular sprouting in human umbilical vein endothelial cells.

DeRita et al. demonstrated that c-Src, IGF-IR, and FAK are contained in prostate cancer cell line-derived EVs [55]. Src is established to stimulate the transcription of *VEGF* and therefore regulate angiogenesis [93]. As discussed in Section 3.4, prostate cancer-derived EVs can also promote angiogenesis in the context of hypoxia [78,79].

### 3.6. Genomic Instability and Mutation

Cancer-derived EVs can induce genomic instability in recipient cells. As previously discussed, EVs may carry mRNA and protein cargo reflecting the status of the donor cell. Chennakirshnaiah et al. demonstrated that EVs released from cancer cells expressing mutant HRAS carried genomic DNA and transferred this material to endothelial cells, which led to abnormal micronuclei formation [94]. Elbakrawy et al. demonstrated that EVs derived from irradiated fibroblast cells enhanced DNA damage in non-irradiated “bystander” fibroblasts [95].

Structural genomic rearrangements are common mechanisms that drive carcinogenesis in prostate cancer [96]. As previously discussed, *TMPRSS2-ERG* gene fusion has been demonstrated in urinary EVs with good correlation to prostatic tissue expression [80].

### 3.7. Resisting Cell Death

Prostate cancer resistance to cell death is a clinical challenge when planning radiotherapy or chemotherapy treatments. The underlying mechanisms are thought to entail a combination of factors including abnormal cell cycle regulation, DNA damage repair, hypoxic and oxidative stress, testosterone signalling, and epithelial–mesenchymal transition [97]. Prostatic EVs are implicated in resisting cell death.

A commonly used chemotherapy agent is docetaxel, which acts by binding to microtubules. Corcoran et al. established docetaxel-resistant variants of the prostate cancer cell lines 22Rv1 and DU145 [72]. They found that EVs expelled from DU145 and 22Rv1 docetaxel-resistant variants (DU145RD and 22Rv1RD) conferred docetaxel resistance in DU145, 22Rv1 and LNCap cells, which may be partly due to EV-mediated MDR-1/P-gp transfer. They found that acquired docetaxel resistance also conferred cross-resistance to doxorubicin (another chemotherapy agent that interacts with DNA intercalation and inhibits the progression of topoisomerase II) as well as anthracycline. In a small pilot study, they isolated EVs from docetaxel-naïve prostate cancer patients (*n* = 6) and age-matched healthy controls (*n* = 6). They found increased proliferation and invasion of cells in the presence of EVs, suggesting they have a causative role. They postulated that the EVs transfer mRNAs, miRNAs, and/or proteins from resistant cells that induce changes in the cellular phenotype of the recipient cells [72].

Changes in expression and mutations in *TP53*, the “guardian of the genome” [98], play a key role in how malignant prostate epithelial cells resist cell death [99]. Metastatic castrate resistant prostate cancer has the highest rates of *TP53* mutation, but this is also seen in primary and castrate-naïve metastatic prostate cancer [100]. miR-27a, transferred between fibroblasts and prostate epithelial cells via EVs, has been shown to reduce the expression of *TP53*. This is an EV-mediated mechanism by which prostate cancer cells can resist cell death and develop chemoresistance.

### 3.8. Deregulating Cellular Metabolism

In order to meet the energy requirements of the high proliferative rate and high growth rate of cancerous cells, a hallmark of malignancy is the deregulation of cellular metabolism [101]. In prostate cancer, this involves acquiring the ability to oxidise citrate to increase ATP production [102] and reprogramming of lipid metabolism [103].

EVs are implicated in the mechanism of metabolic reprogramming through crosstalk between the cancerous cells and cancer associated fibroblasts within the tumour microenvironment. Zhao et al. demonstrated that cancer associated fibroblast-derived EVs can reprogramme the prostate cancer cell metabolism by disabling the mitochondrial oxidative metabolism as well as providing a source of metabolites as molecular cargo [104].

A consequence of high EV production by prostate cancer cells is the increased demand for fatty acids for EV membrane synthesis [105].

### 3.9. Avoiding Immune Destruction

Evasion of destruction by immune cells is a key hallmark of prostate cancer, and evidence from in vitro studies suggests that EVs play a role in this process. Abusamra et al. demonstrated that EVs from LNCaP prostate cancer cells inhibited T cell proliferation and induced T cell apoptosis through a Fas-ligand mechanism, contributing to immune evasion [106].

Mouse knockout model experiments provide further evidence of the role of EVs in the immune evasion processes. Miyazaki et al. demonstrated that *EBAG9* facilitates the escape of prostate cancer from immune surveillance [107]. They demonstrated that spontaneous development of prostate cancer was supressed in a mouse *EBAG9* knockout model. EVs from *EBAG9* overexpressing prostate cancer cells had the potential to facilitate immune escape by inhibition of T cell cytotoxicity and modulating immune-related gene expression in T cells [107].

Kim et al. examined the potential functions of EVs shed from *DIAPH3*-silenced prostate cancer cells [108]. *DIAPH3* is cytoskeletal regulator; the gene encoding it is lost in metastatic prostate cancer with high frequency. The authors found that EVs isolated from *DIAPH3*-silenced cells suppressed proliferation of macrophages and peripheral blood mononuclear cells [108]. EVs isolated from *DIAPH3*-silenced cells contained miR-125a which suppressed AKT1 expression in the mononuclear cells and macrophages.

### 3.10. Tumour-Promoting Inflammation

In prostate cancer, there is an inflammatory response consisting of immune cell infiltration, angiogenesis, and fibroblast proliferation within the tumour microenvironment [109,110]. High levels of inflammatory cytokines (interleukins IL-1, IL-6 and IL-8) promote proliferation and survival [111,112,113]. Many cytokines are released in EV-encapsulated forms [114].

EVs are implicated in the mechanism of sustaining a pro-inflammatory microenvironment. Alaimo et al. demonstrated that TRPM8 is secreted by prostate cells in EVs. When these EVs are taken up, this primes the TLR3/NF-kB mediated inflammatory cascades [115]. Clinically elevated plasma concentrations of EV-associated markers of inflammation (CRP, MCP-1, IFNα2, IL-8, IL-12p70, and MCP-1) have been observed in prostate cancer patients with symptomatic fatigue [116].

### 3.11. Polymorphic Microbiomes as a Prostate Cancer-Enabling Characteristic

Hanahan noted that polymorphic microbiomes provide a distinctive cancer-enabling characteristic, facilitating the acquisition of cancer hallmark capabilities [51]. EVs have been shown to be produced by multiple types of bacteria [36,37,38]. EVs of bacterial origin are frequently detected in EVs isolated from cancer patients [25,117,118,119].

Bacterial sequences have been detected in urinary EVs isolated from patients with prostate cancer [24]. Several of the bacterial sequences identified by RNASeq in the EV fraction belonged to the Anaerobic Bacteria Biomarkers Set (ABBS), with the presence of ABBS in EVs associated with more rapid progression of prostate cancer [24]. The size (20–400 nm) and cargo contents of bacterial EVs include bioactive proteins, toxins, lipids, and nucleic acids and have been shown to be involved in bacteria–host interactions [36,37]. Thus EVs’ functions and associated cancer mechanisms may be linked to multiple origins of EVs, not only from human cells but also bacterial cells.

## 4. Urinary EV-Based Diagnostic Tests for Prostate Cancer

### 4.1. Overview

There is strong evidence that EVs have a role in carcinogenesis, and secreted urinary EVs reflect the dynamic state of the prostate tissue proteome [120]. Urinary EVs and their molecular cargo represent a rich pool of potential biomarkers for prostate cancer diagnosis and progression. Recent years have seen a plethora of studies investigating the relationship between the presence of nucleic acids, proteins, and other molecular markers in prostatic EVs with the presence and grade of prostate cancer. For instance, urinary EV RNA has shown good correlation with prostatectomy tissue RNA detection for *TMPRSS2:ERG* [80]. It is anticipated that molecular analysis of urinary EVs will better represent the multifocal and heterogeneity of disease than traditional needle biopsies [121]. mRNA isolated from EVs has been shown to be more stable than RNA isolated from urinary cells, which again supports an EV-based diagnostic approach [122].

The urinary EV-based diagnostic tests for prostate cancer can be largely classified by the molecular cargo detected in the EV fraction of urine: microRNA, RNA, protein, and other molecules (Supplementary Materials: Table of Studies).

Whilst most of the tests investigated apply urinary diagnostic markers in isolation, some models have incorporated other clinical information such as age and serum PSA to improve the accuracy of their diagnostic model. A limitation observed in many of the studies encountered is that the patient cohorts pre-date the introduction of routine mpMRI, and biopsies were obtained largely by the transrectal ultrasound guided technique, which has now been largely superseded by the transperineal approach in the UK.

### 4.2. Urinary EV RNA-Based Diagnostic Tests

Numerous prostate cancer tests have been developed that utilise RNA expression from the EV fraction of the urine [123,124]. The area under the curve (AUC) of receiver operating curves vary between 0.66 and 0.91 for diagnostic accuracy of any grade of prostate cancer at initial biopsy, and between 0.70 and 0.89 for prediction of Gleason grade group ≥2 disease (Table 2). There is some overlap in the gene probes used in different studies; the most common probe of interest among single and multi-probe RNA classifier-based tests is the *PCA3* gene, which features in 14 studies, and *ERG*, which features in 10 studies.

In terms of translation from scientific concept to use in the clinic, the ExoDx^TM^ Prostate Intelliscore (EPI, formerly known as EXO106) has arguably been the most successful of the EV-based RNA tests to date. EPI was developed in the US and comprises the detection of *ERG* and *PCA3* mRNA relative to SPDEF expression as measured by RT-PCR [125,126,127]. EPI involves collection of the first-catch (i.e., non-DRE) urine, and the sample requires chilled storage until shipment to the central laboratory. Meta-analysis from prospective multisite validation studies generated a pooled AUC of 0.7 for high-grade disease (defined as Gleason grade group 2 or above). The test was validated on patients in the PSA “grey zone” of 2–10 ng/mL scheduled for their initial prostate TRUS needle biopsy. They predicted that using a test cut-off of 15.6 would avoid 23% of all prostate biopsies [127]. At present, EPI is not recommended or available for routine clinical use in the NHS, and the level evidence for its use in individual early detection is considered “weak” in the latest EAU guidelines issued in April 2024 [128].

Tao et al. also developed an RNA-based diagnostic classifier based on the collection of first catch (non-DRE) urine samples. Their test, developed in China, evaluates EV-derived lncRNA expression profiles based on the detection of AC0150987.1, CTD-2589M5.4, RP11-363E6.3. They found that their model generated an AUC of 0.78 and 0.76 for the detection of disease of Gleason grade group 2 or above in two different validation cohorts, respectively [129]. Gan et al. looked at the utility of EV *ERG*, *PCA3*, *PSMA*, *CK19*, and *EpCAM* RNAs in isolation and in combination as a risk model for the identification of the presence of any prostate cancer on initial biopsy. This was based on morning first catch (non-DRE) urine and generated the following AUCs for the presence of any cancer: *ERG* 0.782, *PCA3* 0.783, *PSMA* 0.772, *CK19* 0.731, *EpCAM* 0.739. The combination of *PCA3* and *PSMA* EV RNA generated the highest AUC of 0.870 for any cancer [130]. Whilst these results are both promising, it is worth highlighting that these studies were validated in a predominantly Chinese cohort and may not necessarily extrapolate to similar findings in patient populations of other racial groups. It has long been established that there are racial disparities in the early diagnosis of prostate cancer, and Black men are disproportionately affected [131]. To address this, Kohaar et al. evaluated a two-gene panel (*PCA3*, *PCGEM1*) among a more racially diverse cohort, with a third of the cohort being Black. They found that integrating their two-gene panel with standard-of-care variables (serum PSA, age, and self-reported race) resulted in an AUC of 0.88 for prediction of Gleason grade group ≥2 disease at initial biopsy [132].

Integrating the results of other urinary EV biomarkers with standard clinical information has also shown promise in improving their diagnostic and prognostic accuracy, as demonstrated by work published by our group. Levels of EV mRNAs were combined with clinical parameters and peptides [133], methylation targets [134], and protein levels [135], respectively. Of these, ExoMeth, which combines serum PSA, hypermethylation within the urinary cell pellet as assessed by methylation targets (GSTP1, APC, SFRP2, IGFBP3, IGFBP7, PTGS2), and EV-RNA transcripts (*ERG exons 4–5*, *ERG exons 6–7*, *GJB1*, *HOXC6*, *HPN*, *PCA3*, *SNORA20*, *TIMP4*, *TMPRSS2/ERG fusion*), gave particularly promising results for detection of any cancer on biopsy (AUC 0.91) and stratifying Gleason 3 + 4 or above on initial biopsy (AUC 0.89) [134].

**Table 2:** Urinary EV RNA-based tests with their corresponding AUC of receiver operating curves for detecting Gleason grade group ≥2 prostate cancer on initial prostate biopsy.

Name of Test	Description	Diagnostic Accuracy (AUC)	Reference
Two-gene panel	*PCA3* and *PCGEM1*	0.88 (95% CI 0.81–0.93)	Kohaar, 2021 [132]
3-lncRNA diagnostic model (Clnc)	*AC0150987.1*, *CTD-2589M5.4, RP11-363E6.3*	0.776 (95%CI 0.713–0.838)	Tao, 2023 [129]
EPI score (ExoDx Prostate Intelliscore)	*ERG* and *PCA3* relative to *SPDEF*	0.70 (95% CI 0.65–0.75)	McKiernan, 2018 [126]
EXO106 (EPI ExoDx)	*ERG* and *PCA3* relative to *SPDEF*	0.764 (95% CI 0.691–0.837)	Donovan, 2015 [125]
ExoGrail	*ERG exons 4–5, ERG exons 6–7, GJB1, HOXC6, HPN, PCA3, PPFIA2, SLC12A1, TMEM45B, TMPRSS2/ERG fusion* combined with clinical parameters and EN2 levels	0.84 (95%CI 0.78–0.89)	O’Connell, 2021 [135]
ExoMeth	*ERG exons 4–5, ERG exons 6–7, GJB1, HOXC6, HPN, PCA3, SNORA20, TIMP4, TMPRSS2/ERG fusion* combined with clinical parameters and urine cell DNA methylation data	0.89 (95% CI 0.84–0.93)	O’Connell, 2020 [134]
ExoSpec	*ERG exons 4–5, PCA3, SLC12A1, TMEM45B* combined with clinical parameters and peptides	0.71 (95% CI not reported)	O’Connell, 2022 [133]
GAPT-E score	*GATA2, PCA3, TMPRSS-2*	0.71 (95% CI not reported)	Woo, 2020 [136]
LBXexo score	*PCA3* and *PRAC*	0.736 (95% CI 0.592–0.868)	Ye, 2020 [123]
lncRNA-p21	*lncRNA-p21*	0.663 (95% CI not reported)	Isin, 2015 [137]
Novel urine exosomal lncRNA assay	*PCA3* and *MALAT1*	0.831 (95% CI not reported)	Li, 2021 [124]
Prostate urine risk (PUR)	*AMACR, MEX3A, AMH, MEMO1, ANKRD34B, MME, APOC1, MMP11AR(exons 4–8), MMP26, DPP4, NKAIN1, ERG(exons 4–5), PALM3, GABARAPL2, PCA3, GAPDH, PPFIA2, GDF15, SIM2 (short), HOXC6, SMIM1, HPN, SSPO, IGFBP3, SULT1A1, IMPDH2, TDRD1, ITGBL1, TMPRSS2/ERG fusion, KLK4, TRPM4, MARCH5, TWIST1, MED4, UPK2*	0.77 (95% CI 0.70–0.84)	O’Connell, 2019 [138]

Making the leap from laboratory to clinic is not straightforward. An ideal test would not require a clinic visit for DRE or require complex logistics for sample storage prior to processing. Our group developed the prostate urine risk (PUR) test, which is a risk classifier based on 36 probes [*AMACR*, *MEX3A*, *AMH*, *MEMO1*, *ANKRD34B*, *MME*, *APOC1*, *MMP11AR(exons 4–8)*, *MMP26*, *DPP4*, *NKAIN1*, *ERG(exons 4–5)*, *PALM3*, *GABARAPL2*, *PCA3*, *GAPDH*, *PPFIA2*, *GDF15*, *SIM2 (short)*, *HOXC6*, *SMIM1*, *HPN*, *SSPO*, *IGFBP3*, *SULT1A1*, *IMPDH2*, *TDRD1*, *ITGBL1*, *TMPRSS2/ERG fusion*, *KLK4*, *TRPM4*, *MARCH5*, *TWIST1*, *MED4*, *UPK2*] based on urine collected following DRE. Urine samples were collected from patients from the UK, US and Ireland. PUR-4 status predicted clinically significant prostate cancer with an AUC of 0.77 [138]. We are currently validating this test using a home collection protocol, with samples collected with preservatives that do not require refrigeration or freezing prior to sample processing (which would lend this test well to a prostate cancer screening tool).

### 4.3. Urinary EV miRNA Based Diagnostic Tests

Recently, interest in using microRNAs as a diagnostic tool has sparked, with 10 different urinary EV microRNA-based prostate cancer tests being proposed since 2016 [85,139,140,141,142,143,144,145,146,147,148,149,150]. Groups looked at their role in predicting any grade of prostate cancer upon initial diagnostic biopsy, with AUCs varying between 0.65 and 0.87 where these were reported (Table 3). In terms of individual markers, there was less overlap in probes evaluated than those seen in the larger RNA EV-based diagnostic signatures; however, miR-21 and miR-141 appear in three different studies [84,151,152].

Proving the concept, Foj et al. found that in the EV urine fraction, levels of the microRNAs miR-21, miR-375 and let-7c were significantly upregulated in prostate cancer patients and healthy subjects, as analysed by qRT-PCR [141]. This corresponded to AUCs of 0.713, 0.799 and 0.679, respectively. They also noted that these microRNAs were able to differentiate between the low-D’Amico-risk group and intermediate/high-risk groups.

Koppers-Lalic et al. found that miRNA isoforms (isomiRs) with 3′ end modification were highly discriminatory between urine samples of healthy men versus men with prostate cancer, namely isomiRs of miR-21, miR-204 and miR-375 [151]. Combining these gave an AUC of detecting prostate cancer of 0.821, and when combined with PSA, this increased further to 0.866.

Whilst the majority of work so far has focused on detecting prostate cancer of any grade, future work on microRNA EV-based urinary diagnostics may involve validation of their diagnostic accuracy in distinguishing between Gleason grade groups to triage the need for prostate biopsy. A particularly exciting development was the findings of the miR Sentinel PCa Test, based on microarray data, which evaluated microRNA expression from urinary EVs and classified prostate cancer into indolent and aggressive disease [142]. The test evaluates the expression profile of 130 miRNAs and 66 snoRNAs (small nucleolar RNAs). They found that the Sentinel PCa Test had a sensitivity of 94% and a specificity of 96% for disease of grade group ≥ 3.

Interestingly, one group has developed a urine EV fraction microRNA-based test that predicts metastatic disease and risk of biochemical recurrence, called the Prostate Cancer Metastasis Risk Scoring model (PCa-MRS), based on urinary EV expression profile of miR-21, mi-451, miR-636 expression. Although limited by small study sample size, the PCa-MRS has an AUC of 0.925 for prediction of metastatic disease, with high-scoring patients having worse biochemical recurrence-free survival post radical treatment [84].

### 4.4. Urinary EV Protein-Based Diagnostic Tests

A well-studied protein EV-based urinary marker is the prostate-specific membrane antigen (PSMA), a protein perhaps more popularly known for its utility in nuclear imaging. Wang et al. found that this generated AUC values of 0.876 for the detection of prostate of any grade and AUC of 0.826 for clinically significant prostate cancer. From these data, they concluded that application in a clinical setting could potentially avoid unnecessary biopsies in 41% of cases [143], which represents a significant potential resource saving, whilst missing 0.7% of clinically significant prostate cancer cases (defined as Gleason score ≥ 7).

Other protein-based EV markers include androgen receptor-variant 7 [144], ITGA3 [145], TMEM256 [146], LAMTOR1 [146], CD63 [147], FABP5 [148], and Flotillin 2 [149]. It is worth noting that whilst we typically assume that expression will increase in the cancerous state, transcriptomic profiling has demonstrated that some proteins in the EV urine fraction are negatively regulated, such as cadherin 3 (CDH3). Royo et al. demonstrated that cadherin, which regulates cell–cell adhesion and cellular differentiation, was negatively regulated at the genomic, transcriptional, and epigenetic level in prostate cancer when they profiled urine EVs from prostate cancer patients [150].

## 5. Urinary Prostatic EVs in Active Surveillance of Prostate Cancer

The National Institute for Health and Clinical Excellence (NICE) guideline on prostate cancer [NG131] outlines current recommendations for prostate cancer and treatment in the NHS [9]. The recommended risk stratification tool for men with localised or locally advanced prostate cancer is the Cambridge Prognostic Groups (CPGs). Active surveillance (AS) is offered as a management option for men with CPG 1, 2 or 3 prostate cancer who are fit for radical treatment.

The recommended protocol for AS is as follows:

- Year 1:
  ○ PSA every 3 to 4 months
  ○ DRE at 12 months
  ○ mpMRI at 12 to 18 months
- Year 2 and beyond:
  ○ PSA every 6 months
  ○ DRE every 12 months
  ○ PSA kinetics monitoring, with concerning changes to be re-assessed with mpMRI and/or re-biopsy

In a European meta-analysis incorporating data from over 10,000 men on AS across 12 countries [154], it was found that 15% of men stopped AS within 2 years due to disease progression and treatment. This suggests a need for improved diagnostics to identify which men have a higher risk of early disease progression within risk groups defined by the traditional parameters of PSA, staging, and biopsy histology (which may reflect the misclassification of men with multifocal disease).

Urinary prostatic EV-based diagnostic tests have demonstrated potential for improving the selection of men with prostate cancer for active surveillance. Ramirez-Garrastacho et al. used next-generation sequencing to profile miRNAs extracted from small urinary EVs in a cohort of patients with prostate cancer with ISUP grades 1–3; they then analysed the most promising candidates in a separate 60-patient cohort using RT-qPCR. They found that miR-1290 could differentiate between ISUP grade 1 and 3, miR-320a-3p between ISUP grade 3 and 2, and miR-155-5p between ISUP grades 2 and 1. By combining the miRNAs, they could differentiate between two ISUP grades with an AUC of 0.79–0.88. This is particularly promising in identifying good candidates for AS [155].

Tao et al. recently published the results of their urinary EV long non-coding (lncRNA) classifier, which they found detected grade group 2 or higher prostate cancer and estimated disease progression during AS. They found that the 2-year cumulative incidence of overall AS progression was 19% in patients with low-risk scores compared with 38% in patients with high-risk scores (HR 2.10, 95% CI, 1.16–3.81; *p* = 0.0146) [129]. Their findings will need validation in other patient populations as gene expression signatures have been observed to differ between Asian prostate cancer cohorts and other cohorts such as Caucasian and African American men in Western populations [156].

The Prostate Urine Risk (PUR), developed by our group at the University of East Anglia, is a four-group risk classifier for prostate cancer based upon urine-derived EV RNA. PUR was found to provide additional prognostic information in a cohort of 87 men on active surveillance with 5 years of clinical follow-up. The proportion of PUR-4 dichotomised the cohort into two groups, with differential progression rates of 10% and 60% 5 years after urine collection (HR 8.23, 95% CI 3.26–20.81 [138]. We are currently validating these results in a larger multi-site study using a home urine collection technique as well as evaluating the role of serial sampling during active surveillance for disease monitoring. Due to the role of prostatic EVs in facilitating cancer progression, it is anticipated that EV-based diagnostics may potentially identify disease progression earlier than traditional techniques.

## 6. Future Directions and Conclusions

Prostate cancer continues to increase in incidence, being a significant cause of morbidity and mortality both in the UK and globally [1]. It is imperative that radical treatment decisions are based on accurate and reliable diagnostics for both optimal patient care and resource allocation within stretched healthcare systems.

Urinary EV-based diagnostics is a relatively young and rapidly evolving field that translates multi-disciplinary advances in molecular biology, genomics and bioinformatics into the earlier clinical detection of prostate cancer development and progression. As non-invasive biomarkers, urinary EV-based diagnostics offer the potential for home urine collection [28]. This is particularly attractive in the context of active surveillance, where serial testing over a period of several years is often required. Home collection may bring environmental benefits through reductions in patient travel and improvements in healthcare equity for patients who live in remote areas or struggle to access testing due to socio-economic or cultural factors. This is particularly timely as the NHS is increasingly adopting remote virtual outpatient consultations, which have been demonstrated to have clinical, financial, and environmental benefits in a urological setting [157]. In addition, home collection may lend itself well to a future prostate cancer screening programme, in a similar fashion to the faecal immunochemical test [158].

An area of further investigation and test development may include detection and presence of specific bacteria as markers of aggressive prostate cancer [24], similar to the proposed use of urinary EVs for bacteria detection as diagnostic tests for other cancer types [25,159].

Despite many different urinary EV-based tests being developed and undergoing validation in single patient cohorts, so far, only the ExoDx^TM^ Prostate Intelliscore (EPI) has successfully reached clinical practice. EPI is based upon the expression signature of *ERG*, *PCA3* and *SPDEF* RNA transcripts, is available commercially in the US, and has been granted Breakthrough Device Designation by the US Food and Drug Administration (FDA) [160]. At present, there are no EV-based urine prostate cancer tests in use in the NHS that have been approved by NICE [9], which also reflects the cost-effectiveness of diagnostics as well as clinical utility.

Future research is needed to ascertain the most appropriate points in patient pathways that urinary EV-based biomarkers will fit in, whether as screening tools in asymptomatic men, as biopsy decision triage tools, as risk stratification tools, and/or as disease monitoring tools. Integrating this new prognostic information with prognostic insights from other investigative modalities, particularly MR imaging, will be imperative and require ongoing validation studies and prospective clinical trials in the future.

In conclusion, EVs have roles in intercellular communication, which can alter the prostatic microenvironment for tumorigenesis and accelerate the development of the cancer hallmarks. EVs are an important pool of prostate cancer biomarkers for diagnosis, disease monitoring, and prediction of treatment response. It is anticipated that the next two decades will bring further improvements in diagnostic sensitivity and specificity as well as insights into molecular biological mechanisms of action that can be translated into opportunities in precision uro-oncology.

## Figures and Tables

**Table 1:** Operational terms recommended by extracellular vesicle (EV) terminology MISEV2023.

Operational Term	Description	Example
Physical characteristics	Size with defined ranges	Small EVs (<200 nm)
Biochemical composition	Antigen positivity or biochemical staining	CD63+ EVs
Description of conditions	Cellular conditions	Hypoxic EVs
Cell of origin	Type of cell that EV originates from	Podocyte EVs

**Table 3:** Urinary EV miRNA-based tests with their corresponding AUC of receiver operating curves for detecting the presence of prostate cancer of any grade upon initial prostate biopsy.

Name of Test	Diagnostic Accuracy	Reference
miR-21-5p	0.65 (95% CI 0.477–0.814)	Samsonov, 2016 [152]
miR-30b-3p	0.663 (95% CI 0.011–0.805)	Matsuzaki, 2021 [153]
miR-126-3p	0.664 (95% CI 0.016–5.39)	Matsuzaki, 2021 [153]
miR-501-3p	0.69 (95% CI 0.52–0.85)	Rodriguez, 2017 [139]
miR-196a-5p	0.73 (95% CI 0.56–0.89)	Rodriguez, 2017 [139]
miR-574-3p	0.85 (95% CI 0.736–0.964)	Samsonov, 2016 [152]
miR-141-5p	0.86 (95% CI 0.732–0.994)	Samsonov, 2016 [152]
mi-145 (in combination with serum PSA)	0.863 (95% CI 0.791–0.934)	Xu, 2017 [140]
miR-21, miR-204 and miR-375 (in combination with serum PSA)	0.866 (95% CI not reported)	Koppers-Lalic, 2016 [151]
miR-21, miR-141, miR-375, miR-214 and let-7c	0.872 (95% CI 0.786–0.958)	Foj, 2017 [141]

## Supplementary Materials

The following supporting information can be downloaded at: https://www.mdpi.com/article/10.3390/cancers16091717/s1, Supporting Information: Summary Table of studies. References [80,84,123,124,125,126,127,129,130,132,133,134,135,136,137,138,139,140,141,143,150,151,152,153,161,162,163,164,165,166] are cited in the Supplementary Materials.
